# Network-orchestrated regulation of thymic dysfunction and restoration

**DOI:** 10.3389/fimmu.2026.1896047

**Published:** 2026-09-10

**Authors:** Wenai Sun, Hongbo Chen, Weishan Guo, Qi Guo, Yu Guan, Minghua Li, Gaofeng Qin, Yu Zhang, Wangzhi Wei

**Affiliations:** 1 Liaoning Technology and Engineering Center for Tumor Immunology and Molecular Theranotics, Collaborative Innovation Center for Age-related Disease, Life Science Institute, Jinzhou Medical University, Jinzhou, Liaoning, China; 2 Department of Intensive Care Medicine, Yingkou Central Hospital, Yingkou, Liaoning, China

**Keywords:** androgen receptor (AR), thymic dysfunction, thymic epithelial cell (TEC), thymic homeostasis, thymic microenvironment

## Abstract

Thymic function is governed by an interconnected regulatory network spanning epithelial, stromal, immune and systemic dimensions. This network maintains thymic structure and function through coordinated regulation of epithelial homeostasis, stromal niche integrity, and systemic inputs. Disruption of this network contributes to thymic dysfunction across diverse contexts, including progressive deterioration during aging, whereas regenerative programs identified primarily in models of acute stress or injury represent distinct adaptive responses that can facilitate thymic recovery. Understanding how these regulatory layers differentially shape thymic dysfunction and restoration is essential for developing effective targeting strategies. Progressive attenuation of TEC identity programs, impaired epithelial maturation, reduced proliferative capacity and accumulation of aberrant epithelial states collectively compromise TEC-mediated thymic support during aging. These changes reflect disruption of core epithelial regulatory programs and are accompanied by progressive remodeling of the thymic microenvironment, characterized by chronic inflammatory, oxidative, and metabolic alterations. Together, these processes define a multilayered integrative framework contributing to thymic dysfunction. Importantly, this regulatory landscape exhibits context-dependent plasticity, as specialized epithelial-stromal-immune interactions activated during thymic stress or injury can initiate regenerative programs that support thymic repair, although their contribution to gradual physiological aging remains incompletely defined. These studies indicate that thymic dysfunction and restoration represent distinct outcomes of a shared, context-dependent regulatory network shaped by dynamic cellular interactions and signaling crosstalk. Within this broader regulatory network, sex steroid signaling, including androgen receptor -mediated pathways, represents an important node linking organismal status with thymic homeostasis. Collectively, These insights support a transition from single-target interventions toward combinatorial and context-dependent strategies aimed at restoring thymic function and supporting immune reconstitution.

## 1 Introduction

Aging is accompanied by a progressive decline in immune competence, characterized by diminished responsiveness to infections and vaccinations, impaired tumor immunosurveillance, and increased susceptibility to malignancies and chronic inflammatory disorders (1–4). Collectively referred to as immunosenescence, these changes arise from the interplay between cell-intrinsic aging of immune populations and cell-extrinsic influences imposed by senescent tissues. The latter establishes a chronically dysregulated systemic environment that persistently reshapes immune cell behavior (5, 6). Increasing evidence indicates that immunosenescence is not a simple linear or irreversible decline, but a dynamic and heterogeneous process governed by multi-layered regulatory networks (1). Consequently, effective rejuvenation strategies may benefit from targeting mechanistically defined regulatory nodes rather than indiscriminate immune interventions (1).

Within this framework, the thymus serves as a central hub linking tissue aging with systemic immune competence. Thymic involution begins shortly after birth, accelerates during puberty, and follows a highly conserved trajectory characterized by progressive disruption of epithelial architecture, stromal remodeling, and declining thymopoiesis and thymic output, establishing thymic involution as a major bottleneck contributing to immunosenescence (7, 8).

Recent studies further highlight the clinical relevance of residual thymic function in adulthood. Kooshesh et al. reported that adult thymectomy was associated with increased risks of mortality and cancer incidence (9). Consistently, Bernatz et al. showed that radiographically inferred thymic health was associated with improved overall survival and responses to cancer immunotherapy, suggesting that host thymic activity may represent an additional correlate of therapeutic response, beyond conventional tumor-intrinsic predictors such as PD-L1 and tumor mutational burden (TMB) (10, 11).

Accumulating evidence suggests that thymic involution is not entirely irreversible and partial functional recovery can be achieved under certain conditions (12–15). Advances in spatial profiling and single-cell technologies have further revealed that thymic epithelial and stromal populations are spatially organized into a highly structured microenvironment that coordinates thymocyte development and exhibits region-specific functional plasticity (16, 17). These findings have shifted the conceptual view of thymic aging from a consequence of isolated molecular defects toward a progressive disruption of integrative regulatory networks (18).

**Figure 1:**
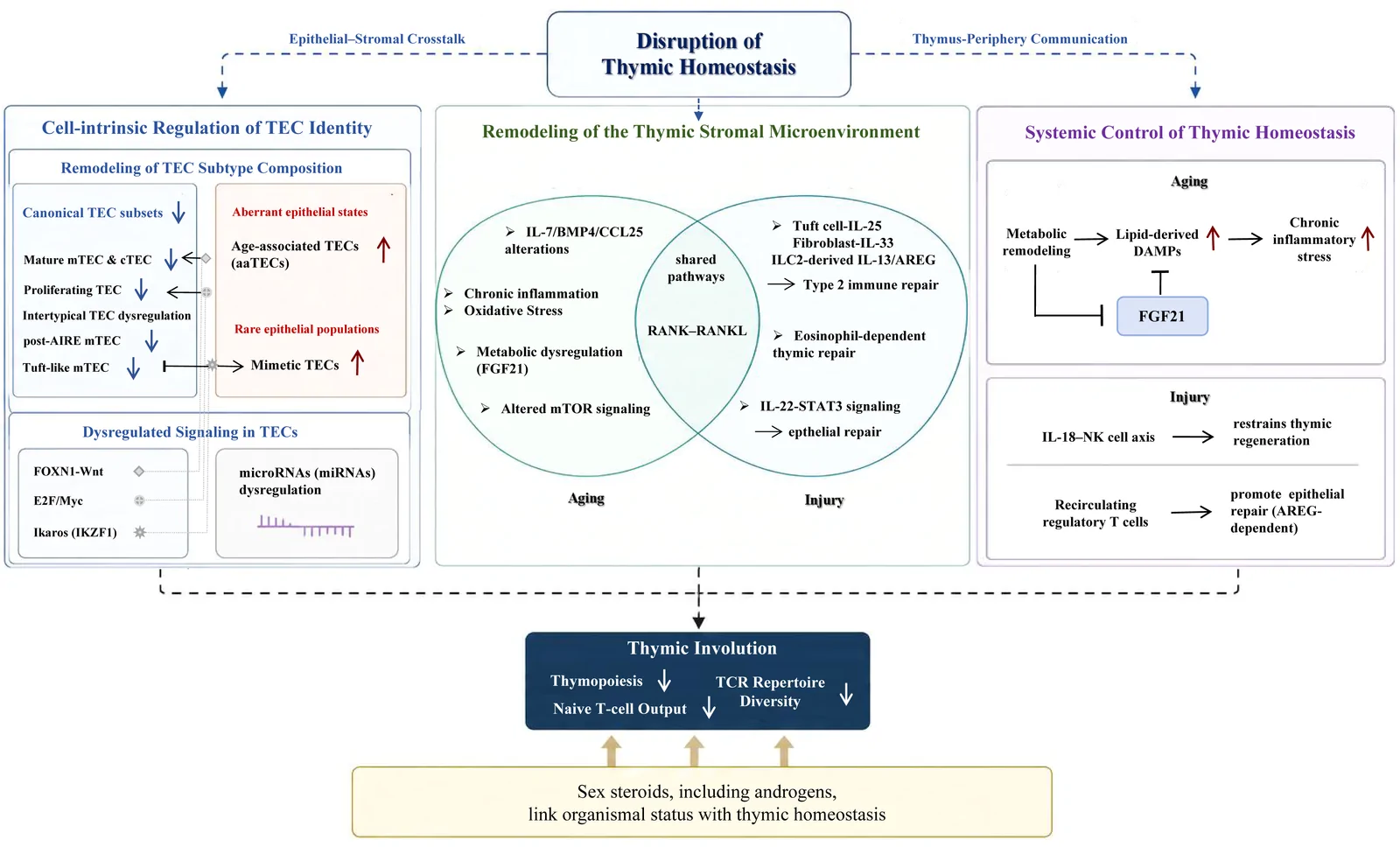
Integrative Regulatory Network underlying Thymic HomeostasisThymic homeostasis is maintained through three interconnected regulatory levels: cell-intrinsic regulation of TEC identity, remodeling of the thymic stromal microenvironment, and thymus-periphery communication. Aging disrupts TEC composition, epithelial signaling, stromal and systemic regulation, leading to progressive thymic dysfunction, whereas acute injury activates distinct regenerative pathways that promote epithelial repair and thymic regeneration. Sex steroid signaling, including androgen signaling, integrate systemic endocrine status with thymic homeostasis. These changes converge to drive thymic involution, resulting in reduced thymopoiesis, naive T cell output and TCR repertoire diversity. Upward and downward arrows denote increased and decreased abundance or activity, respectively. Blunt-ended lines indicate inhibitory or suppressive effects on the corresponding processes.

Importantly, regenerative responses identified in models of acute thymic injury provide valuable mechanistic insights into the latent reparative capacity of the thymus, although they should not be considered equivalent to endogenous drivers of physiological aging. Granadier et al. and Dudakov & van den Brink comprehensively summarized damage-induced regenerative programs, key cellular mediators, and barriers to recovery (19, 20). Conversely, Santamaria & Irla focused on T-cell aging and age-associated thymic involution (13). Although these bodies of work have substantially advanced the field, mechanisms identified in physiological aging and acute injury are frequently discussed together despite arising from different initiating triggers, cellular kinetics, and therapeutic implications. Nevertheless, these distinct biological processes converge on common epithelial, stromal, immune and systemic regulatory pathways that collectively govern thymic integrity, stromal homeostasis, and thymopoietic function. Here, we distinguish these biological contexts while integrating their shared mechanisms into a network-based framework for thymic dysfunction and restoration. Specifically, we organize emerging evidence across three interconnected levels, TEC identity, the thymic microenvironment, and systemic control (**Figure 1**). We then evaluate therapeutic strategies targeting these interconnected layers and discuss their potential to achieve sustained thymic restoration.

## 2 Multilayered disruption of thymic homeostasis

### 2.1 Cell-intrinsic regulation of TEC identity

TECs are central to maintaining thymic architecture and supporting T-cell development, providing both structural scaffolding and essential instructive cues for thymocyte differentiation and selection. TEC identity is therefore fundamental for sustaining thymic homeostasis and the capacity of the thymic epithelium to respond to physiological and pathological challenges. Disruption of TEC identity, including impaired self-renewal and defective lineage progression, compromises epithelial integrity and ultimately limits thymic function (21, 22).

Understanding how TEC identity is maintained requires consideration of their spatial organization within the thymic epithelium. Spatially resolved analyses of the human thymus have refined our understanding of thymic organization, revealing a continuous cortico-medullary axis along which TECs and stromal populations occupy spatially specialized niches. Distinct TEC subsets are distributed along this axis to coordinate thymocyte-epithelial interactions, support T cell development, and shape thymic microenvironments (16). Furthermore, spatial cartography studies indicate that epithelial identity is closely associated with transcriptional programs distributed along this axis, linking spatial organization to epithelial cell states and thymic function (23).

#### 2.1.1 Remodeling of TEC subtype composition

Single-cell profiling has revealed extensive TEC heterogeneity, identifying multiple transcriptionally distinct subsets that collectively sustain epithelial architecture and function (16, 24, 25). These canonical and specialized TEC subsets constitute a dynamic epithelial network that maintains thymic homeostasis. Among these populations, proliferating TECs, characterized by enrichment of cell cycle-associated genes, constitute a proliferative population that declines with age, reflecting reduced epithelial renewal capacity (24). Intertypical TECs, transitional intermediates between precursor-like and mature TEC populations, accumulate with age but exhibit impaired lineage progression, indicating a differentiation bottleneck that limits maturation into functional epithelium (24, 26). Mature TECs undergo functional decline. cTECs, which mediate positive selection via self-MHC recognition, decrease in abundance and exhibit reduced capacity to support thymocyte selection. Similarly, mTECs, responsible for central tolerance through tissue-restricted antigen (TRA) expression, exhibit both quantitative and functional deterioration, including loss of post-AIRE epithelial maintenance programs. Together, these age-associated changes compromise both major checkpoints of effective T-cell development and immune tolerance (27–29). Additional epithelial subsets such as Tuft-like mTECs, which mediate IL-25 dependent signaling, are reduced in aged thymus, potentially compromising thymocyte development (24, 30, 31). Rare epithelial subsets, including neural TECs (nTECs) and structural TECs (sTECs) are implicated in coordinating epithelial-stromal and microenvironmental interactions (24), although their roles in thymic aging remain incompletely defined.

Beyond the remodeling of canonical TEC populations, aging is also associated with the emergence of aberrant epithelial states termed age-associated TECs (aaTECs), including CLDN3^+^ and PDPN^+^ subsets (13). aaTECs exhibit loss of epithelial features, acquisition of stress-associated and epithelial-mesenchymal transition (EMT)-like transcriptional signatures, and formation of thymocyte-depleted epithelial aggregates (13, 22). Rather than stable epithelial lineages, aaTECs likely represent aberrant states arising from dysregulated epithelial maintenance programs. In particular, aaTECs may compete with conventional TEC populations for trophic growth factors without supporting effective thymocyte development (22). Notably, aaTECs undergo substantial expansion following acute thymic stress, further perturbing regenerative signaling pathways and exacerbating injury-induced thymic atrophy by limiting effective epithelial repair (22). Thus, aaTECs represent a pathological convergence linking age-related thymic decline with degeneration after acute damage.

Recent studies have revealed additional layers of TEC heterogeneity beyond the conventional cTEC/mTEC classification. Thymic mimetic epithelial cells represent specialized epithelial populations characterized by peripheral tissue-specific transcriptional programs and molecular features resembling parenchymal cell types, indicating that TEC heterogeneity extends beyond their classical roles in thymic immune education and self-tolerance to encompass broader tissue-mimetic properties (23, 32, 33). Together with age-associated remodeling of canonical TEC subsets and the emergence of aberrant epithelial states, these findings highlight a dynamic view of the thymic epithelium, in which intrinsic epithelial diversification and pathological remodeling contribute to the organization and functional regulation of the thymic microenvironment.

#### 2.1.2 Dysregulated signaling in TECs

TEC identity is governed by signaling networks that coordinate epithelial differentiation, proliferation, and lineage transitions. Disruption of these regulatory pathways contributes to age-associated TEC dysfunction, whereas related signaling mechanisms also influence adaptive responses during acute thymic injury. Among these, FOXN1, a master regulator of TEC lineage differentiation and maintenance, plays a central role in thymic homeostasis. Declining FOXN1 expression with age compromises epithelial self-renewal and thymocyte-supportive functions, thereby contributing to thymic involution (34–36). Consistently, *Foxn1*^lacz^ mutant mice exhibit premature thymic involution accompanied by peripheral T cell alterations and immunosenescence-like phenotypes, providing evidence that TEC-intrinsic FOXN1 deficiency contributes to thymic epithelial dysfunction and associated immune alterations (37). Moreover, FOXN1 is dynamically regulated during injury-induced TEC regeneration. Acute thymic injury or selective depletion of double-positive (DP) thymocytes induces transient FOXN1 upregulation in TECs, accompanied by increased IL-22 production. This injury-responsive FOXN1 upregulation is attenuated upon thymic recovery (38). The FOXN1 regulatory network is further shaped by complementary signaling pathways, including Wnt signaling. Attenuation of Wnt signaling disrupts epithelial progenitor maintenance and promotes EMT-like features and metabolic reprogramming in TECs (15). In this context, altered Wnt pathway activity, including downregulation of Wnt4 and increased LAP2α-associated PPARγ activation, facilitates fibroblast accumulation and promotes adipogenic conversion within the thymic epithelium (13). Beyond the FOXN1-Wnt axis, additional regulators, such as CD147 and IGFBP5 have also been implicated in modulating TEC homeostasis and epithelial dysfunction (39, 40).

Proliferative programs are essential for maintaining TEC integrity and regenerative capacity. TECs exhibit age-associated attenuation of proliferative signaling, characterized by downregulated expression of cell cycle regulators, including E2F target genes, and diminished Myc-dependent transcriptional activity (13). Conversely, restoration of Myc activity in adult TECs enhances epithelial proliferation (12, 13, 41).

Another layer of transcriptional regulation is provided by Ikaros (IKZF1), which is essential for Aire^+^ mTEC homeostasis, thymic mimetic cell diversity, and central tolerance (33). TEC-specific deletion of IKZF1 impairs Aire^+^ mTEC differentiation, leading to loss of tissue-specific antigen expression and impaired central tolerance (33). In addition, Ikaros regulates the transcriptional programs underlying specialized thymic epithelial states, promoting Aire-dependent mimetic populations while restricting the expansion of tuft-like mTECs (33).

At the post-transcriptional level, microRNAs (miRNAs) fine-tune multiple signaling pathways involved in TEC maintenance, proliferation, and function. Altered miRNA profiles contribute to TEC dysfunction by modulating key regulatory programs. For instance, downregulation of miR-181a-5p suppresses TEC proliferation, whereas upregulation of miR-125a-5p reduces FOXN1 expression, accelerating thymic involution (42, 43). More broadly, remodeling of the miRNA landscape converges on key components of the Wnt pathway (44). Collectively, these signaling pathways act within an interconnected network, in which extracellular signals regulate epithelial plasticity, transcriptional programs maintain TEC identity and function, and miRNA-mediated post-transcriptional regulation fine-tunes signaling output. Disruption of this integrative regulatory architecture compromises TEC maintenance and regenerative competence.

### 2.2 Remodeling of the thymic stromal microenvironment

Thymic homeostasis depends on a highly coordinated stromal microenvironment that integrates signals from TECs, developing thymocytes, mesenchymal cells and immune populations. Perturbation of this network contributes to thymic dysfunction during aging, whereas context-dependent remodeling of stromal niches can support tissue repair following acute injury. These processes highlight the critical role of stromal regulation in thymic functional recovery. A key example is the RANK–RANKL axis, where age-associated reduction in RANKL availability impairs mTEC progenitor recruitment and differentiation, contributing to reduced thymic cellularity, decreased frequencies of MHC-II^high^ mTECs, and diminished TRA expression, thereby compromising central tolerance (24, 45). In contrast, following acute thymic injury, such as irradiation, transient induction of RANKL in lymphoid populations contributes to TEC recovery by enhancing epithelial proliferation and survival. RANKL-mediated activation of downstream RANK-LTβR signaling further supports thymic epithelial regeneration, highlighting the importance of stromal-hematopoietic interactions in thymic repair (46).

Alterations in niche-supportive factors, including IL-7, BMP4, and CCL25, disrupt niche signaling networks and compromise the capacity of the thymic microenvironment to support thymocyte development (47–49). In addition, epithelial-supportive IL-22/STAT3 signaling has been implicated in promoting TEC survival and regeneration following thymic injury (50).

Beyond these broadly characterized niche regulatory pathways, recent studies by Nevo et al. reveal that stromal remodeling during injury involves coordinated epithelial-stromal-immune interactions, in which tuft cells act as upstream sensors of epithelial stress and initiate immune activation through tuft cell-derived IL-25 and fibroblast-derived IL-33 signaling, thereby engaging ILC2 responses. Fibroblasts act as active niche components functionally coupled to tuft cells, together establishing a coordinated pro-regenerative microenvironment. Within this network, ILC2s serve as a central effector hub that integrates stromal-derived cues into downstream IL-13- and amphiregulin (AREG)-mediated outputs, promoting restoration of TEC homeostasis and thymic function (51). Consistent with this framework, Cosway et al. describe a parallel effector module in which eosinophils represent a component of regenerative type 2 immune circuits associated with thymic tissue recovery. Eosinophil depletion impairs thymic epithelial architecture, supporting a role for eosinophils as downstream effectors of type 2 immune-mediated regenerative responses (52).

While these injury-induced programs highlight the regenerative potential of the thymic microenvironment, aging is associated with progressive stromal remodeling characterized by chronic inflammatory, oxidative, and metabolic alterations. Elevated pro-inflammatory cytokines, including IL-1β, IL-6, and TNF-α, impair TEC function and accelerate tissue deterioration (13). Accumulation of reactive oxygen species (ROS)-induced DNA damage further compromises epithelial stability (53, 54), whereas pharmacological targeting of mitochondrial ROS has been shown to partially restore thymic structure, supporting redox imbalance as a key contributor to stromal dysfunction.

Metabolic regulation represents an additional layer contributing to age-associated changes in thymic stroma. FGF21 has emerged as an important metabolic regulator of thymic homeostasis, modulating mTORC1/2 activity in cTECs through mTEC-derived growth factor signaling to support epithelial function (55), while enhancing stromal production of IL-7, SCF, and IGF-1 to reinforce local niche activity. In addition, FGF21 suppresses age-associated adipogenic remodeling through PPARγ downregulation, limiting ectopic adipocyte accumulation and fibroadipogenic cell expansion (56). Notably, although adipocytes progressively accumulate during aging, they may also serve as an additional source of FGF21 with thymus-supportive effects, suggesting a complex feedback relationship between adipogenic changes and metabolic regulation within the aging thymus (56, 57). Together, these findings support a multi-source, stromal-targeted regulatory network in which FGF21 integrates signals from epithelial, mesenchymal, and adipogenic compartments, linking metabolic regulation with stromal homeostasis during thymic aging.

### 2.3 Systemic control of thymic homeostasis

Regulation of thymic homeostasis involves bidirectional thymus-periphery communication, integrating metabolic, innate immune and adaptive immune signals. Disruption of these systemic inputs contributes to age-associated thymic decline, whereas distinct immune and metabolic responses can modulate thymic repair following acute injury. Together, these interactions highlight that thymic function is regulated within a broader systemic context, with distinct cues shaping thymic deterioration and restoration.

Consistent with this concept, recent single-cell analyses of paired human thymus and peripheral blood further reveal coordinated remodeling of T-cell developmental trajectories across thymic and peripheral compartments (18). Age-associated decline in thymic output is accompanied by alterations in peripheral T cell states, emphasizing that thymic aging occurs within a systemic regulatory landscape rather than occurring in isolation.

Among these systemic regulatory inputs, metabolic alterations represent a major component of age-associated thymic dysfunction, linking energy status to thymic homeostasis. During aging, accumulation of lipid-derived metabolites acts as a source of lipotoxic damage-associated molecular patterns (DAMPs) that activate innate immune pathways and promote chronic inflammatory states, thereby contributing to impaired thymic function and altered immune regulation (58, 59). FGF21 represents an important metabolic regulator within this axis. By enhancing lipid utilization and stimulating perithymic brown adipose tissue activity, FGF21 reduces the burden of lipid-derived DAMPs and alleviates lipid-associated inflammatory stress (59). This metabolic regulation has been associated with enhance thymic output, linking systemic energy metabolism with thymic functional recovery (59).

Beyond chronic metabolic alterations associated with aging, systemic immune signals also actively regulate thymic responses following acute injury. Innate immune signaling introduces an additional regulatory layer during tissue repair. Granadier et al. demonstrated that, IL-18 activates NK cells through cytotoxic programs that, while contributing to host defense and tumor immune surveillance, may lead to TEC damage (60). This IL-18–NK cell axis therefore acts as a damage-responsive checkpoint imposing a functional brake on repair responses (60). Notably, although IL-18 is being explored as a cancer immunotherapy agent due to its ability to induce type 1 immune responses, these findings highlight potential off-target effects on thymic homeostasis (60).

In contrast, adaptive immune populations represent another component of systemic regulation that can promote thymic regeneration following injury. Recirculating regulatory T cells migrate back to the thymus following peripheral activation and promote epithelial repair and thymopoiesis (61). Distinct from *de novo* thymic Tregs, recirculating Tregs can be identified using the Rag2GFP reporter system, with Rag2GFP^-^ Tregs preferentially localized to the thymic medulla. Computational analyses reveal a transcriptional program associated with tissue repair in these cells. Lemarquis et al. demonstrated that recirculating Tregs promote thymic epithelial repair through AREG-dependent signaling (61).

Together, these findings demonstrate that systemic immune and metabolic cues exert context-dependent effects on thymic homeostasis (**Figure 1**), influencing both age-associated decline and responses to thymic stress. Within this framework, endocrine signals provide an additional layer of control that integrates organismal status with thymic function. Among these endocrine regulators, androgen signaling emerges as an important node within the broader regulatory network controlling thymic homeostasis.

## 3 Androgen signaling in thymic homeostasis and immune regulation

### 3.1 Thymus-dependent effects of androgen signaling

Androgen signaling is mediated predominantly through the androgen receptor (AR) expressed in TECs, establishing the thymic epithelium as the principal target of androgen action (62, 63). Consistent with this epithelial-centered mode of regulation, TEC-specific AR knockout (ARKO) models exhibit marked thymic enlargement, increased TEC abundance, and enhanced thymopoiesis, supporting a role for androgen signaling as a physiological brake on TEC homeostasis (62, 63). In contrast, thymocyte-restricted effects appear to be comparatively limited (64). Given the substantial molecular and functional heterogeneity of TEC subsets (24, 65), AR signaling is likely to exert context-dependent effects across distinct epithelial populations. Collectively, these findings provide a mechanistic explanation for the temporal association between the pubertal surge in sex steroids and accelerated thymic involution (66), and suggest that androgen signaling may also contribute to sex-related differences in immune responsiveness (67, 68).

Androgen deprivation has been shown to alleviate androgen-mediated constraints on epithelial homeostasis (64). This is accompanied by expansion of both cortical and medullary epithelial compartments, together with partial restoration of epithelial-stromal architecture. In preclinical models, these structural changes are accompanied by enhanced thymopoiesis and increased naive T cell output (64). Clinical studies suggest that ADT is associated with increased recent thymic emigrants (RTEs) and enhanced T-cell responses in patients with prostate cancer (8, 69), providing indirect evidence consistent with enhanced thymic export. However, these findings are heterogeneous across studies and patient populations, and the magnitude, durability, and direct attribution of thymic restoration remain incompletely defined. Together, the evidence suggests that androgen signaling contributes to the regulation of epithelial quiescence and responsiveness to regenerative signals (22).

### 3.2 Broader immune consequences of androgen signaling

Androgen signaling extends beyond the thymus to influence peripheral T-cell immunity through effects on TCR repertoire diversity and immune surveillance capacity. These effects have important implications for anti-tumor immunity, as androgen-dependent regulation of T cell homeostasis can influence CD8^+^ T cell functional states within the tumor microenvironment (70). However, the relative contribution of restored thymic output versus direct AR-dependent reprogramming of peripheral T cells and the tumor microenvironment to these antitumor effects remains difficult to disentangle.

Sex differences in tumor immune surveillance further illustrate the broader impact of androgen signaling within a multilayered regulatory network involving sex chromosomes, sex hormones, and tumor-immune interactions (71). Within this framework, AR signaling modulates CD8^+^ T cell differentiation and function through both systemic regulation of T cell homeostasis and cell-intrinsic transcriptional programs. Accordingly, AR blockade reshapes the tumor microenvironment toward a more immunostimulatory state and enhances responsiveness to immune checkpoint blockade (70). These systemic effects are reflected by sex-biased patterns of CD8^+^ T-cell dysfunction, with male T cells exhibiting stronger exhaustion-associated signatures and impaired effector function, thereby potentially contributing to compromised tumor immune control (72).

Androgen-dependent immune regulation also intersects with broader age-associated immune dysfunction programs that influence anti-tumor immunity. In melanoma, senescence-associated transcriptional programs have emerged as important features associated with tumor-infiltrating lymphocyte fitness and responsiveness to immune checkpoint blockade (73). Although senescence and exhaustion represent distinct biological processes, their transcriptional and functional convergence suggests a continuum of dysfunctional T cell states rather than completely separate phenotypes. Consistently, patients who fail to respond to immunotherapy exhibit enrichment of senescence-associated signatures across multiple lymphoid populations, including CD4^+^ T cells, CD8^+^ T cells, B cells, and NK cells (73). This senescent immune landscape is characterized by impaired cytotoxic capacity, disrupted immune communication, and a senescence-associated secretory phenotype (SASP)-driven immunosuppressive microenvironment, collectively contributing to tumor immune dysfunction and therapeutic resistance (73).

Androgen signaling represents one component of a broader endocrine regulatory network that influences immune and thymic homeostasis. Other hormonal pathways, including estrogen, progesterone and glucocorticoid signaling, also contribute to the regulation of immune function and thymic remodeling (74–77). The integration of these hormonal signals with immune and stromal regulatory networks helps shape thymic function and peripheral immune competence, providing a framework for developing rational rejuvenation strategies.

## 4 Approaches toward thymic restoration and immune reconstitution

Therapeutic strategies for thymic restoration have increasingly focused on improving thymic immune competence. These approaches have demonstrated varying degrees of success in enhancing thymic structure, thymopoietic function, or peripheral immune reconstitution. However, improved peripheral immune reconstitution should not be interpreted as direct evidence of thymic rejuvenation. This highlights a fundamental challenge in the field: to date, there is a lack of clinically established strategy capable of directly and specifically restoring human thymic function in a mechanistically-validated and endpoint-definitive manner. Indeed, previous studies have discussed the strategies by integrating findings from distinct biological levels, while insufficiently distinguishing direct evidence of thymic rejuvenation from indirect evidence reflected by peripheral immune outcomes. To address this conceptual gap, we evaluate current approaches according to their target compartments, mechanistic basis, and functional endpoints. This framework provides a more realistic assessment of the current translational landscape and highlights the need for clinically validated thymus-specific endpoints to establish effective strategies for human thymic rejuvenation.

### 4.1 Interventions targeting the thymic microenvironment

Given the central role of the thymic microenvironment in thymic involution, strategies aimed at restoring epithelial niches have emerged as promising approaches to improve thymic function (**Table 1**). A recombinant FOXN1 fusion protein incorporating the N-terminal domain of CCR9 has been engineered to enable thymus-targeted delivery. In aged mice, administration of this fusion protein, which is preferentially taken up by TEC, enhances TEC maintenance and function, thereby promoting thymopoiesis and increasing T cell output (78).

**Table 1:** Approaches toward thymic restoration and immune reconstitution.

Major strategy	Intervention type	Approach	Targeted compartments / Cells	Thymus-specific effects	Peripheral immune outcomes	Evidence levels	Limitations
Thymic Microenvironment- targeted Strategy	Thymus-targeted delivery	Recombinant FOXN1-CCR9 Fusion Protein	TECs	↑ TEC abundance; ↑ thymocyte cellularity and ETPs; enhanced thymopoiesis	↑ peripheral T cell pool	Preclinical: aged mouse models	No human data; long-term delivery efficiency, safety, and clinical feasibility remain to be established.
	Growth factor-based intervention	KGF/FGF7	TECs	↑ thymic size and cellularity; ↑ TEC proliferation and organization; ↑ thymopoiesis; ↑ thymic T cell output	↑ peripheral T cell pool; ↑ T cell-dependent antigen-specific antibody responses	Preclinical: aged mouse models; Clinical: phase I/II trials (NCT01712945; NCT02356159)	Context-dependent and variable clinical outcomes; observed benefits primarily reflect peripheral immune reconstitution, with limited evidence for direct restoration of human thymic function.
	Cytokine-based intervention	Interleukin 22 (IL-22)	TECs	↑ TEC proliferation and survival	↑ T cell reconstitution; tissue repair and inflammatory control	Preclinical: adult mouse injury models; Clinical: phase I/II trial (NCT02406651)	Observed benefits primarily reflect peripheral T cell reconstitution, with limited evidence for direct restoration of human thymic function.
	Signaling-based intervention	RANK–RANKL	TECs	↑ thymic medulla and Aire+ mTEC cellularity; ↑ mTEC differentiation	↑ peripheral T cell pool; ↑ T cell-mediated immune responses	Preclinical: aged and injury-induced mouse models	Potential systemic toxicity due to effects on bone metabolism (e.g. osteoporosis risk)
	Cellular intervention	TEPCs or PSC-derived TECs	TECs	↑ TEC proliferation; ↑ thymopoiesis;	↑ peripheral T cell reconstitution; ↑ peripheral naive T cells;	Preclinical: adult and aged mouse models; nude mouse models	Variability in differentiation/reprogramming efficiency; uncertainty regarding graft persistence; long-term stability of induced epithelial identity
		FOXN1-reprogrammed embryonic fibroblasts (iTECs)	TECs	↑ thymic size and cellularity; ↑ thymopoiesis; ↑thymocyte negative selection; ↑ TEC organization;	↓ senescent or autoreactive T cells; ↑ T cell-mediated immune responses	Preclinical: aged mouse models	
	Bioengineering intervention	3D thymic organoids & decellularized thymus scaffolds	TECs	↑ epithelial architecture; ↑ TEC and thymocyte density/cellularity; ↑ thymopoiesis and T cell differentiation	↑ peripheral T cell pool	Preclinical: adult and nude mouse models	Long-term functional validation and in vivo integration remain to be established
Developing Thymocyte- targeted Strategy	Hematopoietic progenitor expansion	Flt3 ligand (Flt3L)	Flt3⁺ lymphoid progenitors	↑ thymopoiesis; ↑ thymocyte development ↑ thymic output	peripheral T-cell reconstitution; ↑ TRECs	Preclinical: adult mouse models	limited evidence for direct restoration of human thymic function
	Progenitor-based cellular approach	Adoptive transfer of in vitro- derived proT cells	T cell progenitors	↑ thymopoiesis; ↑ T cell development;	↑ TCR repertoire diversity	Preclinical: adult mouse models	Translational feasibility has yet to be established
	Chemokine-mediated progenitor recruitment	CCL25/CCR9; CCL21/CCR7	Hematopoietic progenitors	↑ thymic seeding; ↑ intrathymic migration; ↑ thymopoiesis	——	Preclinical: adult mouse models	Clinical feasibility remains to be established.
	Cytokine-based intervention	Interleukin 7 (IL-7)	T cell progenitors and developing thymocytes	↑ thymocyte development; ↑ thymopoiesis; ↑ thymocyte survival and proliferation	↑ circulating naive and memory T cells; ↑ peripheral T-cell homeostasis; ↑ CD8⁺ T cell responses	Preclinical: aged mouse models; Clinical: phase I trial (NCT00684008)	Observed benefits primarily reflect peripheral immune reconstitution, with limited evidence for direct restoration of human thymic function.
		Interleukin 21 (IL-21)	T cell progenitors and developing thymocytes	↑ thymopoiesis; ↑ thymic output (↑ RTEs)	↑ peripheral T cell pool; ↑ T cell-mediated immune responses; improved vaccine responsiveness	Preclinical: aged mouse models;	Clinical feasibility remains to be established.
Androgen Signaling - targeted Strategy	Androgen deprivation therapy (ADT)	GnRH/LHRH agonist-based castration (e.g., Leuprolide, Goserelin, Triptorelin)	HPG axis	↑ thymic size and architecture; ↑ thymopoiesis; ↑ thymic output	↑ TRECs; ↑ peripheral T cell pool; ↑ TCR repertoire diversity	Preclinical: aged mouse models; aged non-human primates (baboons)	Direct clinical evidence for structural or functional restoration of the human thymus remains limited
		GnRH/LHRH antagonist-based castration (e.g.., degarelix, relugolix)	GnRH/LHRH receptor	↑ thymocyte cellularity; ↑ thymopoiesis;	↑ TRECs; ↑ peripheral T cell pool	Preclinical: adult/aged mouse models	
		AR antagonist-based blockade (e.g. flutamide,bicalutamide, nilutamide, enzalutamide, apalutamide, darolutamide)	Androgen receptor (AR)	↑ thymic size and cellularity; ↑ thymocyte survival; positive selection;	Context-dependent effects on peripheral T-cell immunity	Preclinical: AR-knockout and aged mouse models	Potential off-target immune modulation and therapeutic resistance; direct clinical evidence for structural or functional restoration of the human thymus remains limited

TEC, thymic epithelial cell; EPT, early thymic progenitor; TEPC, thymic epithelial progenitor cell; PSC, pluripotent stem cell; iTEC, induced thymic epithelial cell; TCR, T cell receptor; RTEs, recent thymic emigrants; Flt3L, Fms-like tyrosine kinase 3 ligand; KGF, keratinocyte growth factor; GnRH, gonadotropin-releasing hormone; LHRH, luteinizing hormone-releasing hormone; HPG axis, hypothalamic–pituitary–gonadal axis.The symbol “↑” indicates an increase, enhancement, or promotion, representing a positive effect, whereas “↓” indicates the opposite.

Growth factor-based approaches such as keratinocyte growth factor (KGF/FGF7), an epithelial cell-specific growth factor, have been investigated for their ability to modulate TEC compartments and improve thymic function. In aged mice, KGF treatment increases thymic size and cellularity, enhances TEC abundance and organization, and promotes thymopoiesis and thymic T cell output. These effects are associated with increased peripheral T cells and improved T cell-dependent antigen-specific antibody responses, supporting the potential of KGF as a strategy for counteracting age-associated thymic decline (79, 80). However, clinical studies have reported variable outcomes, including limited or even reduced recovery of peripheral naive CD4^+^ T cells, circulating RTEs, and T-cell receptor excision circles (TRECs) (NCT01712945) (81). In contrast, a recent phase I/II trial (NCT02356159) demonstrated that high-dose administration of palifermin (KGF) was feasible and promoted immune reconstitution, as evidenced by increased frequencies of CD4^+^ RTEs and naive CD4^+^ T cells, together with reduced CD4^+^ regulatory T cells (Tregs) and a lower Treg-to-naive CD4^+^ T cell ratio (82). These findings suggest that optimized KGF-based strategies may enhance post-transplant T cell recovery; however, direct evidence demonstrating restoration of human thymic function remains limited.

IL-22 promotes thymic epithelial repair via the JAK1/TYK2–STAT3 signaling pathway (83). In a phase II clinical study (NCT02406651), IL-22 administration demonstrated an acceptable safety profile and showed potential to enhance post-transplant T cell reconstitution, accompanied by effects on tissue repair and inflammatory control, supporting its role as an epithelial-protective cytokine (84).

The RANK–RANKL pathway is a potent inducer of TEC differentiation through regulation of the key mTEC transcription factor Aire. It restores the thymic epithelial compartment by promoting TEC maturation and expansion, particularly of Aire^+^ mTECs (45, 83). However, the translational application of RANKL is complicated by its intrinsic coupling of thymic epithelial regulation with bone metabolism. Given its role in osteoclastogenesis, systemic administration may disrupt skeletal homeostasis and increase the risk of bone loss. This dual role represents a major limitation of systemic RANKL therapy, as thymic benefits may be inherently linked to off-target skeletal effects. Accordingly, successful clinical translation will likely require tissue-targeted strategies that spatially and temporally restrict RANKL signaling to minimize systemic side effects (85).

Cell-based strategies provide complementary avenues for TEC restoration, including fetal thymic epithelial progenitor cell (TEPC)-based thymus reconstitution, as well as neonatal- or pluripotent stem cell-derived TEC-like approaches with the potential to restore thymic function (83). Meanwhile, FOXN1-reprogrammed embryonic fibroblasts acquire a TEC-like transcriptional program characterized by activation of thymic epithelial identity genes and suppression of fibroblast lineage signatures, conferring TEC-like functional properties (86, 87). Following intrathymic delivery, these engineered cells engraft within the thymic niche and facilitate epithelial reconstruction, thereby supporting thymopoiesis and T-cell differentiation in aged models (86, 87). However, these strategies remain at the preclinical stage. Key translational challenges include variability in reprogramming efficiency, uncertainty regarding graft persistence and long-term stability of induced epithelial identity, which must be addressed prior to clinical translation (87).

Finally, bioengineering strategies aim to reconstruct thymic architecture using three-dimensional organoids and biomaterial scaffolds that recapitulate TEC-thymocyte interactions within a defined microenvironment. By incorporating key stromal and signaling cues, these engineered systems provide a platform for generating functional artificial thymus-like structures while restoring epithelial organization and supporting thymopoiesis and T-cell differentiation (88–90).

### 4.2 Interventions targeting developing thymocytes

T cell development relies on continuous replenishment by hematopoietic progenitors. Accordingly, strategies aimed at increasing the availability of hematopoietic progenitors have emerged as potential approaches to facilitate thymic restoration (**Table 1**). Preclinical studies have shown that administration of Flt3 ligand expands bone marrow lymphoid progenitors, thereby enhancing thymopoiesis through increased progenitor supply and altered the distribution of thymocyte subsets (83, 91).

Alternatively, progenitor-based cellular therapies seek to directly replenish thymus-seeding progenitors (92). One strategy involves the adoptive transfer of *in vitro*-derived proT cells generated using OP9-DL1 co-culture systems (93, 94). These early T-lineage progenitors, typically characterized by a CD34^+^CD7^+^/^++^ phenotype, efficiently home to and engraft the thymic niche, thereby supporting thymopoiesis and T cell development (94). Among them, CD5^+^ proT2 cells exhibit superior thymic reconstitution capacity, highlighting functional heterogeneity among early T-lineage intermediates (95). Importantly, because these cells lack surface TCR expression, they require less stringent histocompatibility matching (96). Collectively, these progenitor-based strategies restore the early cellular input required for thymopoiesis and support thymic reconstitution.

Chemokine networks coordinate hematopoietic progenitor recruitment and intrathymic trafficking of developing thymocytes, potentially compensating for limited progenitor availability (83). Among these, the CCL25-CCR9 and CCL21-CCR7 axes play critical roles in regulating progenitor homing and intrathymic migration (97). Preclinical studies have shown that modulation of these signaling axes enhances thymic seeding and cellular migration within the thymus, thereby promoting thymopoiesis.

IL-7 signals through its receptor complex to activate PI3K and JAK–STAT pathways and serves as a non-redundant survival factor supporting thymocyte development (83). IL-7 deficiency or TEC-specific deletion of IL-7 expression results in depletion of both αβ and γδ T cell compartments and developmental arrest at the DN2/DN3 transition (83, 98). Targeted delivery of IL-7 through engineered IL-7–CCR9 fusion proteins increases developing thymocyte numbers and enhances CD8^+^ T cell responses in aged animals (13). In contrast, recombinant human IL-7 (CYT107) administration primarily promotes peripheral T cell expansion, including naive and effector memory T cell subsets, accompanied by increased TCR repertoire diversity and improved peripheral T cell homeostasis and function (NCT00684008) (99). However, direct clinical evidence demonstrating restoration of human thymic function remains limited. Similarly, IL-21 enhances thymopoiesis and increases circulating recent thymic emigrants (RTEs) in aged mice, suggesting improved thymic output and vaccine responsiveness (100).

### 4.3 Interventions targeting androgen signaling

Given the well-established influence of sex steroids on thymic homeostasis, androgen deprivation therapy (ADT) has been extensively explored as a strategy to alleviate age-associated thymic decline (**Table 1**) (69). In aged mouse models, castration promotes recovery of early thymocyte progenitor populations, enhances thymocyte cellularity and thymopoiesis, improves thymic architecture and partially corrects age-associated alterations in thymocyte proliferation and apoptosis (63, 69, 101). Similarly, androgen deprivation in aged baboons resulted in thymic enlargement accompanied by expansion of CD3^+^ and CD4^+^/CD8^+^ thymocyte populations and increased RTEs, providing supportive evidence for enhanced thymus-associated function in a non-human primate model (102). In clinical studies, elderly patients receiving ADT for prostate cancer have exhibited increased thymic output-associated biomarkers, suggesting enhanced thymus-associated immune recovery, although direct evidence for structural or functional restoration of the human thymus remains limited (69, 103).

Despite these potential benefits, systemic androgen deprivation is associated with metabolic and endocrine adverse effects that may limit its long-term clinical applicability. Moreover, improvements in peripheral immune parameters, including thymic output-associated measures, should not be interpreted as evidence of thymic rejuvenation in the absence of direct assessments of thymic structure and function. These limitations underscore the need for more rigorous approaches to distinguish genuine thymic restoration from secondary peripheral immune reconstitution. To date, androgen modulation has been explored both as a standalone strategy and in combination with immune checkpoint blockade (104, 105) or thymus-targeted interventions, such as epithelial growth factors (e.g., KGF) (106), yielding encouraging effects on antitumor immunity and peripheral immune reconstitution. However, clinical evidence demonstrating structural or functional restoration of the human thymus remains lacking. Most available studies have focused on peripheral immune recovery or oncologic outcomes, whereas validated thymus-specific structural and functional endpoints have rarely been incorporated as endpoints. Establishing such endpoints to directly evaluate human thymic restoration therefore remains an important translational challenge that warrants further investigation.

## 5 Conclusions

Thymic dysfunction is increasingly recognized as a consequence of coordinated disruption across multiple regulatory layers rather than isolated cellular or molecular defects. Under physiological conditions, thymic homeostasis is maintained through the dynamic integration of thymic epithelial cell identity, stromal niche integrity, and systemic regulatory cues. Disruption of this interconnected network ultimately compromises thymic function and immune competence. This network-oriented perspective provides not only a more comprehensive understanding of the mechanisms underlying thymic dysfunction but also a conceptual foundation for developing rational restoration strategies.

Guided by this framework, a broad spectrum of therapeutic approaches has emerged, targeting complementary components of the thymic regulatory network. Collectively, these interventions have demonstrated encouraging efficacy in enhancing thymopoiesis and improving immune function in preclinical models. However, their clinical translation remains limited. Although peripheral immune recovery and antitumor efficacy have frequently been reported, convincing evidence demonstrating structural and functional restoration of the human thymus remains lacking. Importantly, improvements in peripheral immune parameters, including thymic output-associated measures, should not be interpreted as definitive evidence of thymic restoration in the absence of direct assessments of thymic structure and function.

Future progress will therefore depend not only on developing more effective rejuvenation strategies but also on establishing standardized, thymus-specific endpoints for therapeutic evaluation. Integrating multimodal assessments, including thymic imaging, measurements of thymopoiesis and thymic output, and high-resolution immune profiling into clinical studies will be essential for distinguishing genuine thymic restoration from secondary peripheral immune reconstitution. Establishing such a network-guided, endpoint-driven framework will enable more rigorous evaluation of emerging therapies, facilitate their clinical translation, and ultimately accelerate the development of effective therapies for restoring thymic function.
